# A protective role for type I interferon signaling following infection with *Mycobacterium tuberculosis* carrying the rifampicin drug resistance-conferring RpoB mutation H445Y

**DOI:** 10.1371/journal.ppat.1012137

**Published:** 2024-04-11

**Authors:** Suhas Bobba, Kuldeep S. Chauhan, Sadia Akter, Shibali Das, Ekansh Mittal, Barun Mathema, Jennifer A. Philips, Shabaana A. Khader

**Affiliations:** 1 Department of Molecular Microbiology, Washington University School of Medicine, St. Louis, Missouri, United States of America; 2 Division of Infectious Diseases, Department of Medicine, Washington University School of Medicine, St. Louis, Missouri, United States of America; 3 Department of Microbiology, University of Chicago, Chicago, Illinois, United States of America; 4 Department of Epidemiology, Columbia University Mailman School of Public Health, New York, New York, United States of America; New Jersey Medical School, UNITED STATES

## Abstract

Interleukin-1 (IL-1) signaling is essential for controlling virulent *Mycobacterium tuberculosis* (*Mtb*) infection since antagonism of this pathway leads to exacerbated pathology and increased susceptibility. In contrast, the triggering of type I interferon (IFN) signaling is associated with the progression of tuberculosis (TB) disease and linked with negative regulation of IL-1 signaling. However, mice lacking IL-1 signaling can control *Mtb* infection if infected with an *Mtb* strain carrying the rifampin-resistance conferring mutation H445Y in its RNA polymerase *β* subunit (*rpoB*-H445Y *Mtb*). The mechanisms that govern protection in the absence of IL-1 signaling during *rpoB*-H445Y *Mtb* infection are unknown. In this study, we show that in the absence of IL-1 signaling, type I IFN signaling controls *rpoB*-H445Y *Mtb* replication, lung pathology, and excessive myeloid cell infiltration. Additionally, type I IFN is produced predominantly by monocytes and recruited macrophages and acts on LysM-expressing cells to drive protection through nitric oxide (NO) production to restrict intracellular *rpoB*-H445Y *Mtb*. These findings reveal an unexpected protective role for type I IFN signaling in compensating for deficiencies in IL-1 pathways during *rpoB*-H445Y *Mtb* infection.

## Author summary

*Mycobacterium tuberculosis* (*Mtb*) is the causative agent of tuberculosis (TB) and is responsible for 10 million active cases worldwide annually. The acquisition and dissemination of drug resistance to front-line antibiotics have stifled efforts to control and combat TB. Understanding the immune responses elicited during *Mtb* infection is imperative to develop novel therapeutics that bolster protective responses and limit detrimental processes. Recently, we found that mice do not require the inflammatory interleukin-1 (IL-1) signaling pathway to control *Mtb* infection if the infecting bacterial strains carry a specific drug resistance-conferring mutation. Instead, here we show that a different signaling pathway, type I interferon (IFN), mediates protection against drug-resistant mutant *Mtb* infection in the absence of IL-1 signaling. We also found that the primary producers of type I IFN during drug-resistant *Mtb* infection were recruited innate cells, namely monocytes and macrophages. The downstream impacts of IFN signals were varied, with macrophages restricting bacterial replication while other immune cell functions were likely impaired by IFN signaling. Our study provides new insights into the different host requirements for protection against infection by various *Mtb* strains and highlights the unexpected roles for type I IFN signaling during *Mtb* infection.

## Introduction

*Mycobacterium tuberculosis* (*Mtb*) is estimated to cause 10 million active cases of tuberculosis (TB) annually [1]. Global dissemination of drug resistance to front and second-line antibiotics limits efforts to contain and combat this pervasive disease [2–7]. Alternatively, to avoid the toxicity associated with anti-TB chemotherapies, researchers are developing host-directed therapeutics to better augment the host immune response to *Mtb* infection and effective vaccines that protect against TB disease. These strategies require a thorough understanding of the host-pathogen interactions and the immune events correlated with protective responses to *Mtb* infection.

Substantial efforts have been made to understand important immune cell populations and signaling pathways necessary for host protection and survival during *Mtb* infection [8–14]. Interleukin-1 (IL-1) signaling is essential for protection during *Mtb* infection, as hosts with defective IL-1 signaling experience immunopathology and succumb to infection [15,16]. Conversely, type I interferon (IFN) signaling is thought to be detrimental during *Mtb* infection through antagonism of IL-1 signaling and induction of maladaptive inflammatory processes [17–19]. In humans, type I IFN signaling is associated with TB disease progression from controlled *Mtb* infection [20–23]. We previously found that the presence of a drug resistance-conferring mutation, *rpoB*-H445Y, can alter host-pathogen interactions such that IL-1 signaling is not necessary to control drug-resistant (*rpoB*-H445Y) *Mtb* infection [7,24]. Additionally, cytokine production is altered during infection of macrophages with *rpoB*-H445Y *Mtb in vitro* and *in vivo* [25], with heightened type I IFN and limited IL-1 production. The *rpoB*-H445Y *Mtb* strain contained an increased abundance of the long-chained lipid phthiocerol dimycocerosate (PDIM) in its cell wall. Treatment of wildtype (*wt*) *Mtb*-infected BMDMs with purified PDIM from *rpoB*-H445Y *Mtb* was sufficient to drive altered cytokine production [24]. However, we have not determined the IL-1-independent protective pathways that led to the control of this *rpoB*-H445Y *Mtb* infection.

In the current study, we demonstrate that, in contrast to what is seen with wild type (*wt) Mtb* infection, type I IFN signaling has a protective role during *rpoB*-H445Y *Mtb* infection. While a global loss of either IL-1 or type I IFN signaling alone did not affect *rpoB*-H445Y *Mtb* replication *in vivo*, mice with deficient IL-1 signaling were susceptible to *rpoB*-H445Y *Mtb* infection when they also lacked type I IFN signaling. Increased *rpoB*-H445Y *Mtb* burden was associated with an increase in inflammatory cell influx and inflammation. Furthermore, recruited myeloid cells, such as monocytes and macrophages, were the source of type I IFN during *rpoB*-H445Y *Mtb* infection. Interestingly, the role of type I IFN signaling in host protection depended upon cell type, such that it was protective in LysM^+^ cells but detrimental in CD11c^+^ cells. *In vitro* experiments suggested that both type I IFN and IL-1 signaling led to nitric oxide (NO) production, which limited *rpoB*-H445Y *Mtb* growth. Our results highlight the complex role of type I IFN signaling and that host protective mechanisms can be markedly different during infection with two closely related bacterial strains. Insights from this work could be used to suggest potential targets for future avenues of study in the development of host-directed therapies against drug-resistant *Mtb*.

## Results

### Type I IFN signaling mediates protection in the absence of IL-1 signaling in *rpoB*-H445Y *Mtb* infection

**Fig 1:**
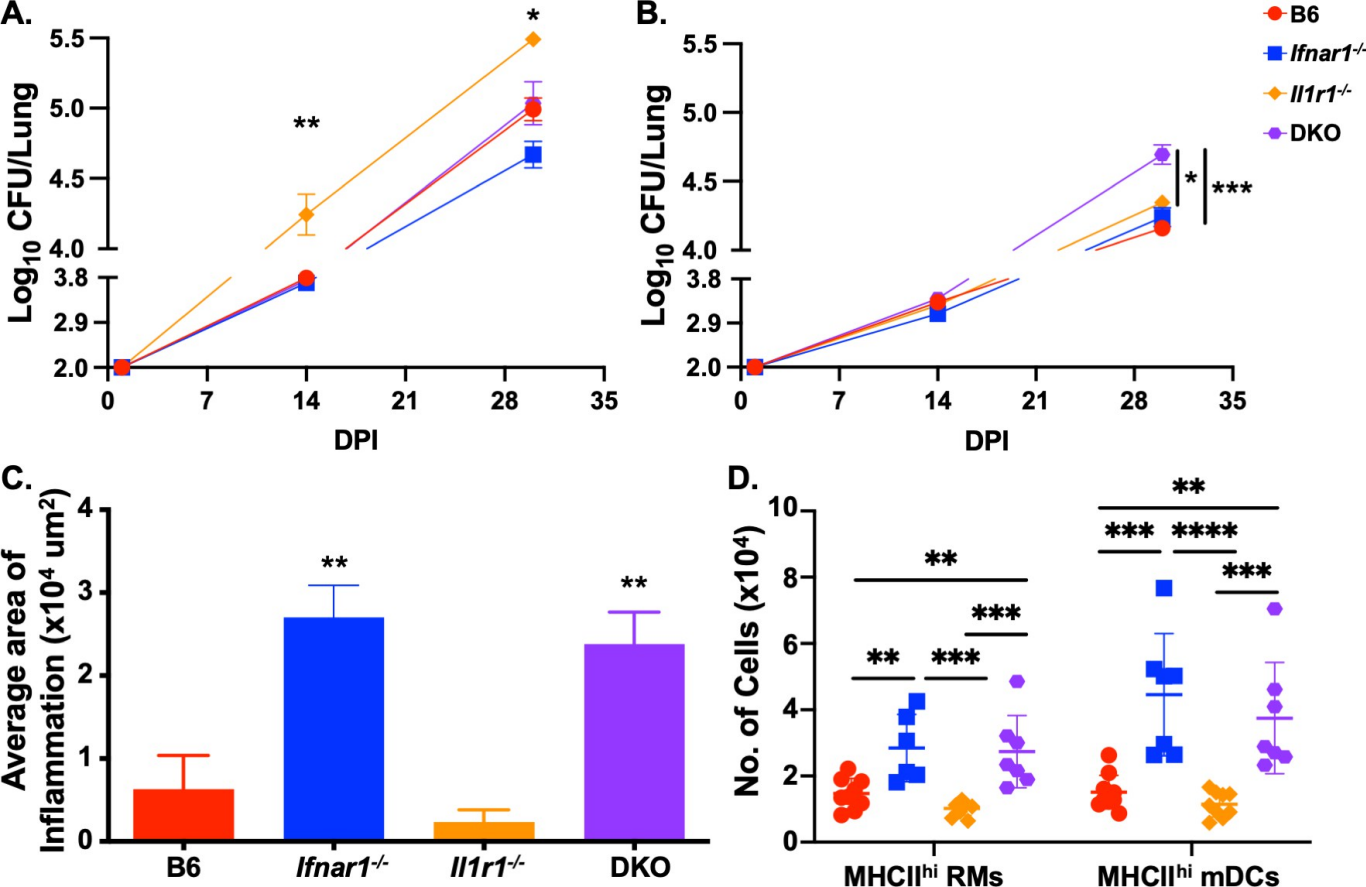
Type I interferon signaling is protective in the absence of interleukin-1 signaling in *rpoB*-H445Y *Mycobacterium tuberculosis* infection. C57Bl/6 (B6), *Ifnar1*^*-/-*^, *Il1r1*^*-/-*^, and *Ifnar1*^*-/-*^ *Il1r1*^*-/-*^ [double knockout (DKO)] mice were aerosol infected with a low dose of *wt* or *rpoB*-H445Y *Mtb*. Lung *Mtb* burden was evaluated at 14- and 30-days following A) *wt* or B) *rpoB*-H445Y *Mtb* infection. C) Formalin-fixed, paraffin-embedded (FFPE) lung sections were stained with hematoxylin and eosin (H&E), and the total inflammatory areas were quantified after *rpoB*-H445Y *Mtb* infection at 30 days post-infection (dpi). D) Using flow cytometry, the total numbers of lung MHCII^hi^ RMs and mDCs were determined at 30 dpi after infection with *rpoB*-H445Y *Mtb*. The data shown represent the means ± SD of seven to ten mice per experiment. The data were evaluated for normality using the Shapiro-Wilk Test and passed (p-value > 0.05). Two-way ANOVA was used for A and B, and One-way ANOVA was used for C and D, with Tukey’s multiple comparisons test. Significant differences are indicated with asterisks (*, p-value ≤ 0.05; **, p-value ≤ 0.01; ***, p-value ≤ 0.001; ****, p-value ≤ 0.0001) by appropriate statistical tests. One of three independent experiments shown.

IL-1 signaling is essential in controlling *wt Mtb* infection [15,18]. However, this signaling pathway is dispensable when mice are infected with *rpoB*-H445Y *Mtb* [24], a drug-resistant but non-isogenic *Mtb* strain derived from *wt Mtb* [25], suggesting that there are alternate signaling pathways mediating protection. To determine if type I IFN signaling was protective in the absence of IL-1 signaling against *rpoB*-H445Y *Mtb in vivo*, we generated mice lacking both IL-1 and type I IFN receptors and evaluated their susceptibility to *wt* or *rpoB*-H445Y *Mtb* infection. As expected, *Il1r1-/-* mice were unable to control *wt Mtb* infection, with elevated lung burden at 14- and 30-days post-infection (dpi), relative to C57BL/6 (B6) mice (Fig 1A), whereas loss of *Ifnar1* alone has no impact on *Mtb* burden. As before, following *wt Mtb* infection [18,20], the increase in lung burden caused by loss of *Il1r1* was reversed in mice lacking both *Ifnar1* and *Il1r1* [double knockout (DKO)] (Fig 1A). An increase in the susceptibility of *Il1r1*^*-/-*^ mice was accompanied by an increase in inflammatory damage in the lungs of *wt Mtb* infected (S1A Fig), but this inflammatory damage did not significantly decrease in the DKO mice where Mtb control was evident. We also observed increased numbers of recruited macrophages (RMs) expressing high levels of Major Histocompatibility Complex Class II (MHCII^hi^), a surrogate marker for activation, of neutrophils, and of MHCII^hi^ monocytes in the lungs of *wt Mtb*-infected *Il1r1*^*-/-*^ mice (S1B Fig). In the infected DKO mice where susceptibility was reversed, only the numbers of neutrophils were significantly decreased. We found no differences in the numbers of cytokine-producing, activated (CD44^hi^) T lymphocyte populations between the infected groups (S1C and S1D Fig). A different pattern of susceptibility was apparent when we infected the mice with *rpoB*-H445Y *Mtb*. We observed higher bacterial burdens only in the DKO mice at 30 dpi (Fig 1B), while loss of either IL1 or type I IFN signaling alone did not impact lung *rpoB*-H445Y *Mtb* burden. The increased bacterial load in the DKO *rpoB*-H445Y *Mtb*-infected lungs also coincided with increased lung inflammation (Fig 1C) and increased numbers of lung MHCII^hi^ RMs and MHCII^hi^ myeloid dendritic cells (mDCs) in the DKO mice at 30 dpi (Fig 1D). However, there were no differences in the numbers of neutrophils, MHCII^hi^ monocytes, MHCII^hi^ alveolar macrophages (AMs), and cytokine-producing, CD44^hi^ T lymphocyte populations between the DKO mice and B6 and *Il1r1*^*-/-*^ mice (S2A–S2E Fig). As previously noted [25], loss of *Ifnar1* alone was sufficient to drive an increase in the numbers of these cellular populations and the amount of inflammation during *rpoB*-H445Y *Mtb* infection. Thus, while type I IFN signaling is dispensable globally for control of *wt* and *rpoB*-H445Y *Mtb* infection, in the absence of IL-1 signaling, type I IFN signaling is necessary for immunity against *rpoB*-H445Y *Mtb*.

### Similar type I IFN transcriptional pathways are induced during *wt* and *rpoB*-H445Y *Mtb* infections but result in divergent infection outcomes

**Fig 2:**
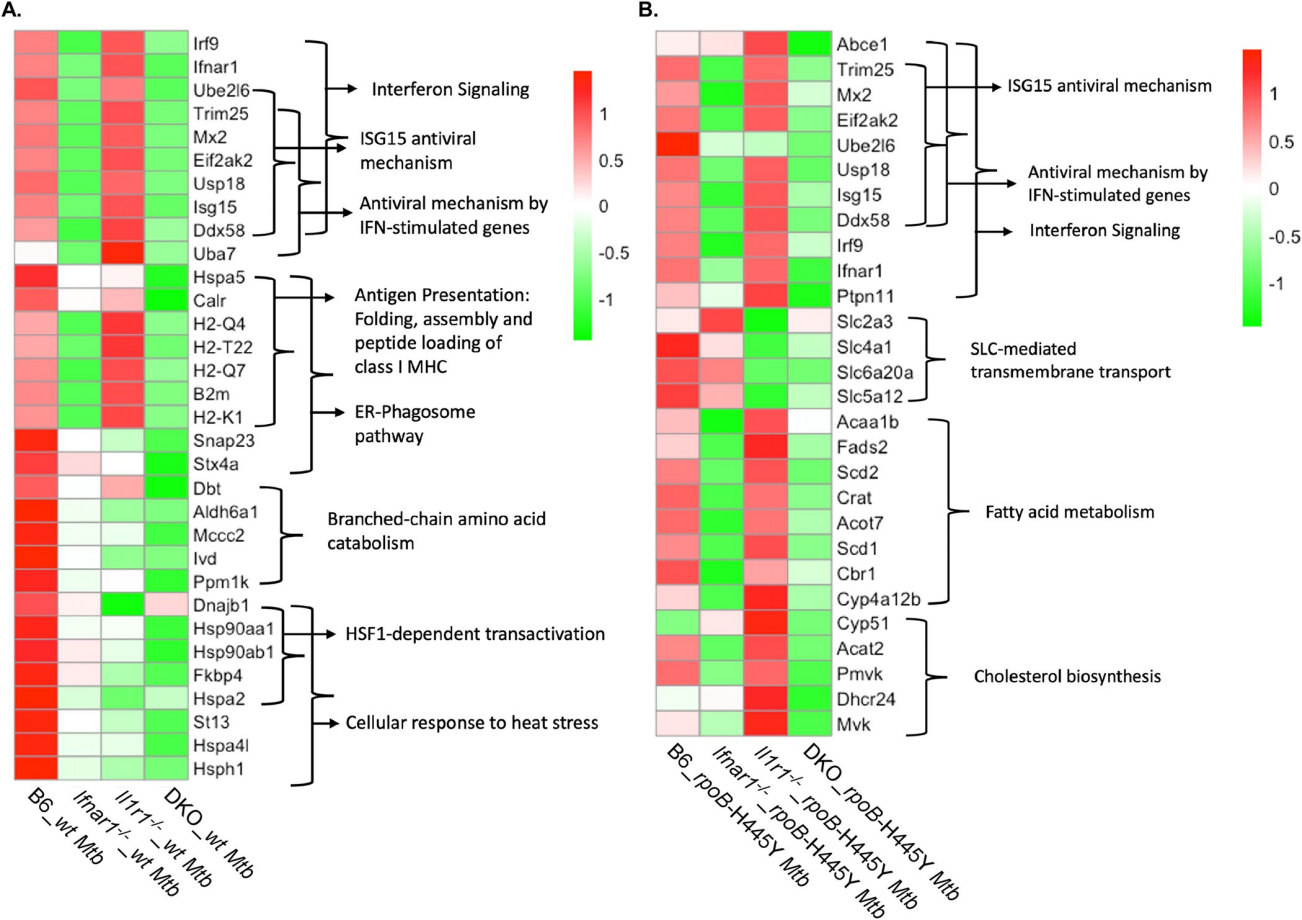
Type I IFN signaling signatures are common across *wt* and *rpoB*-H445Y *Mtb* infections. C57Bl/6 (B6), *Ifnar1*^*-/-*^, *Il1r1*^*-/-*^, and *Ifnar*^*-/-*^ *Il1r1*^*-/-*^ [double knockout (DKO)] mice were aerosol infected with a low dose of *wt* or *rpoB*-H445Y *Mtb* and sacrificed at 14 dpi. RNA was extracted from homogenized lung tissue and sequenced. Heatmaps displaying average expression levels of top differentially expressed genes (DEGs) across mouse genotypes after A) *wt* or B) *rpoB*-H445Y *Mtb* infection with annotated pathways (four to six mice per group). Significant DEGs related to the corresponding reactome pathways are visualized using heatmaps(|log_2_FC| >0, adjusted p-value < 0.05). Average expression (normalized) of selected genes on log_2_ scale are shown.

Type I IFN signaling has been thought to be detrimental during *wt Mtb* infection based upon its association with the progression of TB patients towards active disease and its ability to antagonize the protective IL-1 signaling pathway [18,20,22]. To discern the roles of type I IFN signaling during both *Mtb* infections, we determined the lung transcriptional landscape in response to either infection at 14 days post-infection, an early timepoint before significant differences in lung *rpoB*-H445Y *Mtb* burden were apparent in the gene-deficient mice. We identified the most differentially expressed genes (DEGs) across groups and categorized them by pathways (Fig 2). In the lungs of mice infected with *wt Mtb*, genes related to IFN signaling were upregulated in the B6 and *Il1r1*^*-/-*^ mice compared with *Ifnar1*^*-/-*^ and DKO mice (Fig 2A). Specific analysis of the type I IFN transcriptional signature revealed downregulation of genes in this pathway in the *Ifnar1*^*-/-*^ and DKO mice when compared to the B6 and *Il1r1*^*-/-*^ mice during *wt Mtb* infection (S3A Fig). Additionally, genes involved in antigen presentation via MHC I, such as *H2-q4* and *H2-t22*, were induced by type I IFN signaling during *wt Mtb* infection at this early timepoint. We also observed induction of genes involved in branched chain amino acid catabolism (*Aldh6a1* and *Ivd*), phagocytosis [26] (*Snap23* and *Stx4a*), endoplasmic reticulum (ER) stress [27,28] (*Hspa5* and *Calr*), and cellular response to heat stress (*Hsp90aa1* and *St13*) specifically in the B6 mice infected with *wt Mtb* relative to the single knockout and DKO mice (Fig 2A). We observed enrichment of DEGs in pathways involved in type I IFN signaling during *wt Mtb* infection in both B6*-* and *Il1r1*^*-/—*^infected mouse groups, although they differed in their ability to control infection. Together, our data suggest that decreased co-expression of type I IFN-induced genes with genes involved in metabolism and stress could drive detrimental does not determine the outcomes of *wt Mtb* infection.

In the lungs of mice infected with *rpoB*-H445Y *Mtb*, we also observed that DEGs related to IFN signaling were upregulated in B6 and *Il1r1*^*-/-*^ mice, whereas these pathways were downregulated in the *Ifnar1*^*-/-*^ and DKO mice (Fig 2B). Indeed, the type I IFN transcriptional signature was downregulated in both the *Ifnar1*^*-/-*^ and DKO mouse groups when compared with either *wt* or *Il1r1*^*-/-*^ mice following *rpoB*-H445Y *Mtb* infection (S3B Fig). Interestingly, expression of some interferon-stimulated genes (ISGs), such as *Abce1* and *Ptpn11*, was higher in the lungs of *Il1r1*^*-/-*^ mice than in B6 mice after *rpoB*-H445Y *Mtb* infection. Another IFN-related gene, *Ube2l6*, decreased in expression in the *rpoB*-H445Y *Mtb* infected *Il1r1*^*-/-*^ mice lungs relative to infected B6 counterparts (Fig 2B). These findings suggest that IL-1 signaling plays a complex role in the regulation of IFN-related genes. Coincident with the upregulation of IFN-related genes in B6 and *Il1r1*^*-/-*^ *rpoB*-H445Y *Mtb*-infected mice, genes involved in metabolic pathways, such as fatty acid metabolism and cholesterol biosynthesis were upregulated. In contrast, mice lacking IL-1 signaling exhibited decreased expression of genes, such as *Slc2a3* and *Slc4a1*, that are part of the solute carrier (SLC)-mediated transmembrane transport family. Therefore, our data indicate that the loss of *rpoB*-H445Y *Mtb* control observed in DKO mice was associated with the downregulation of various cellular processes that could be triggered by either type I IFN or IL-1 signaling. Many IFN-related genes, such as *Isg15*, *Ifnar1*, and *Mx2*, were induced following either *wt* or *rpoB*-H445Y *Mtb* infection. In contrast, genes such as *Abce1* and *Ptpn11* were only upregulated following *rpoB*-H445Y *Mtb* infection, whereas *Uba7* was upregulated only during *wt Mtb* infection. Furthermore, *Ube2l6*, which exhibited differential regulation in *Il1r1*^*-/-*^ and B6 mice during *rpoB*-H445Y *Mtb* infection, was upregulated in both groups of mice during *wt Mtb* infection. So, while we observed broad induction of many IFN signaling-related genes during both *Mtb* infections, some IFN-related genes were differentially induced across *wt* and *rpoB*-H445Y *Mtb* infections.

### CD11b^+^ myeloid cells produce type I IFN, while LysM^+^ cells respond to type I IFN following *rpoB*-H445Y *Mtb* infection

**Fig 3:**
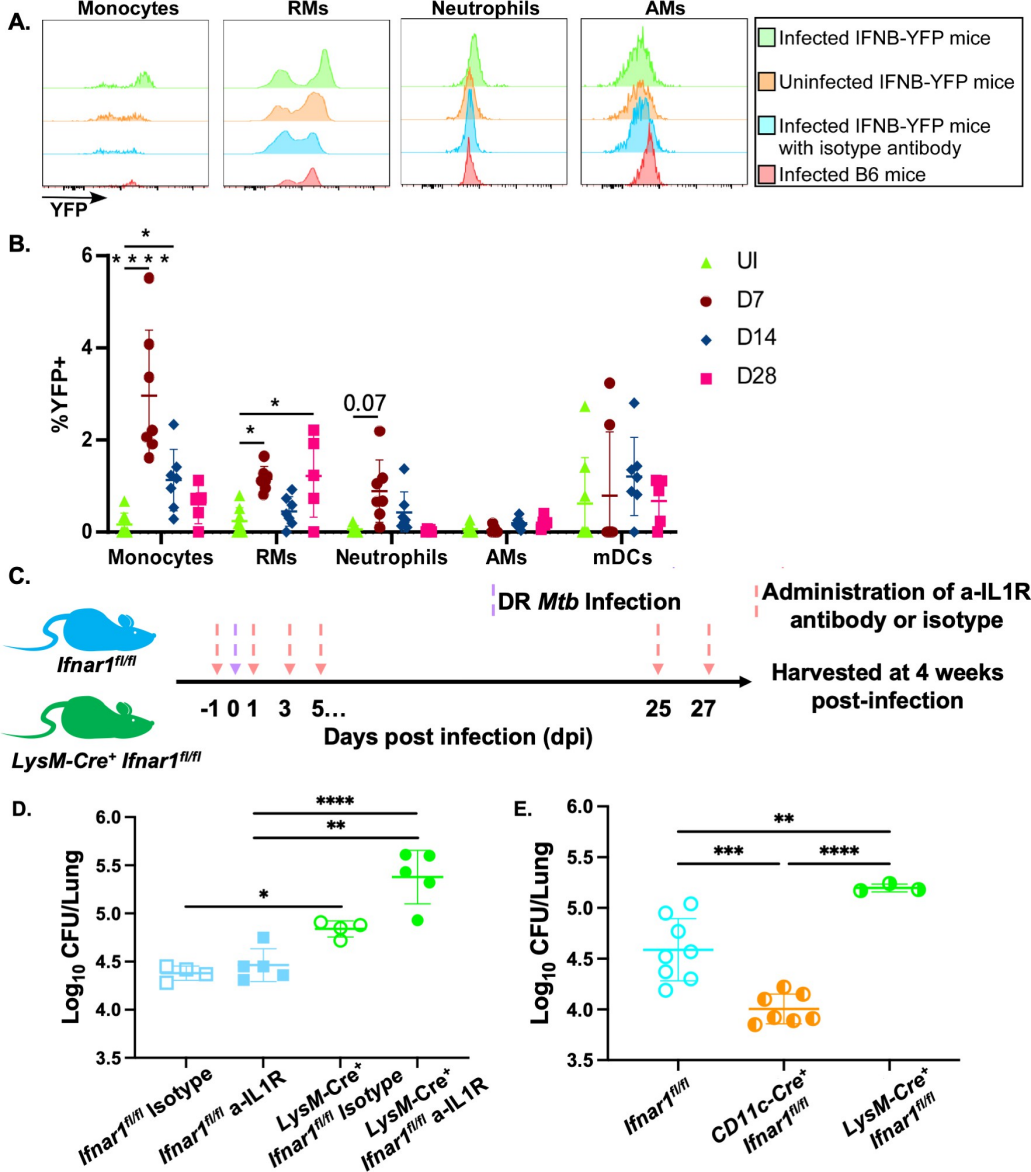
Type I IFN producers are CD11b^+^ myeloid cells, while LysM^+^ cells respond to type I IFN following *rpoB-*H445Y *Mtb infection*. IFNβ-YFP mice were aerosol infected with *rpoB*-H445Y *Mtb* and sacrificed at the indicated timepoints along with uninfected controls. A) Representative histograms for various subsets [Monocytes (left), Recruited Macrophages (RMs) (middle left), Neutrophils (middle right), and Alveolar Macrophages (AMs) (right)] depicting YFP expression by intracellular antibody staining (green) compared to controls [uninfected IFNβ-YFP mice stained with intracellular antibody (orange), infected IFNβ-YFP mice stained with intracellular isotype antibody (blue), and infected B6 non-reporter mice stained with intracellular antibody (red)]. B) The frequencies of YFP^+^ cells were determined by flow cytometry following sacrifice of *rpoB*-H445Y *Mtb*-infected IFNβ-YFP mice at 7, 14, and 28 dpi, along with uninfected controls. C) Experimental scheme for the *LysM-Cre Ifnar1*^*fl/fl*^ mice with the purple arrow indicating aerosol infection with *rpoB*-H445Y *Mtb* and the pink arrow indicating administration of a-IL1R or isotype antibodies i.p. D) Lung *Mtb* burden was determined at 28 dpi. E) *LysM*-*Cre*^+^ *Ifnar1*^fl/fl^ (semi-closed green circles) and *Cd11c*-*Cre*^+^ *Ifnar1*^*fl*/fl^ mice (semi-closed orange circles), along with littermate controls (open blue circles), were infected with *rpoB*-H445Y *Mtb*, and bacterial burden was determined in the lungs at 28 dpi. The data shown represent the means ± SD of three to eight mice per experiment. The data were evaluated for normality using the Shapiro-Wilk Test and passed (p-value > 0.05). Two-way ANOVA and One-way ANOVA, with Tukey’s multiple comparisons tests, were used for B and D-E, respectively. Significant differences are indicated with asterisks (*, p-value ≤ 0.05; **, p-value ≤ 0.01; ***, p-value ≤ 0.001; ****, p-value ≤ 0.0001) by appropriate statistical tests. One-way ANOVA. Two independent experiments shown.

Type I IFN is rapidly produced by many cell types in response to infection [21,29]. We used IFNβ-YFP reporter mouse strain, in which cells that produce IFN β also co-express YFP and are readily detectable [30–32] (Fig 3A), and found that type I IFN production was induced in various myeloid lung populations following infection with *rpoB*-H445Y *Mtb* (Fig 3B). As early as 7 dpi, *rpoB*-H445Y *Mtb* infection lead to significantly higher accumulation of IFN-producing lung monocytes and RMs as compared with uninfected controls. No discernable production of type I IFN in AMs, neutrophils, or any non-immune (CD45^-^) population was detected at this timepoint. Plasmacytoid dendritic cells (pDCs), a major producer of type I IFN during bacterial or viral infection [14,32], were not readily detected in our analysis. We then tracked type I IFN production in *rpoB*-H445Y *Mtb* infection at later timepoints (Fig 3B). Lung monocytes were also the primary cell type expressing type I IFN production at 14 dpi, and RMs were the major cellular population that was producing type I IFNs at 28 dpi. Taken together, our data indicate that the main sources of type I IFN production in *rpoB*-H445Y *Mtb* infection are CD11b^+^ myeloid cells, namely monocytes and RMs.

Loss of control of *rpoB*-H445Y *Mtb* infection in mice lacking type I IFN and IL-1 signaling was accompanied by an influx of inflammatory myeloid cells, and the primary producers of type I IFN throughout *rpoB*-H445Y *Mtb* infection were myeloid cells. To test whether myeloid cells were also responding to type I IFN in *rpoB*-H445Y *Mtb* infection in the absence of IL-1 signaling, we generated *LysM-Cre Ifnar1*^*fl/fl*^ mice (deletion of the type I IFN receptor on LysM-expressing myeloid cells [33]), infected these mice with *rpoB*-H445Y *Mtb*, and also treated them with IL-1 blocking antibodies or isotype controls (Fig 3C). As we observed in the *rpoB*-H445Y *Mtb*-infected *Il1r1*^*-/-*^ mice, blocking of IL-1 signaling with antibody did not impact lung bacterial burden in the *Ifnar1*^*fl/fl*^ mice (Fig 3D). But, similar to the *rpoB*-H445Y *Mtb*-infected DKO mice, loss of the type I IFN receptor in *LysM-Cre*^*+*^ *Ifnar1*^*fl/fl*^ mice treated with IL-1 signaling blocking antibody led to an increase in lung *Mtb* burden. Unexpectedly, loss of the type I IFN receptor in the LysM-expressing cells alone led to increased *rpoB*-H445Y *Mtb* lung burden (Fig 3D). The increased susceptibility in *LysM-Cre*^*+*^ mice infected with *rpoB*-H445Y *Mtb* correlated with increased pulmonary inflammation (S4A Fig) and increased accumulation of lung MHCII^hi^ RMs and mDCs (S4B Fig). Together, our results suggest that type I IFN signaling is necessary in LysM^+^ cells to mediate control of r*poB*-H445Y *Mtb* infection but dispensable when deleted globally as in the *Ifnar1*^*-/-*^ mice. To better understand this apparent discrepancy, we assessed the impact of the loss of type I IFN signaling in CD11c-expressing cells, which include AMs and DCs (Fig 3E). *Cd11c-Cre*^*+*^ *Ifnar1*^*fl/fl*^ mice better controlled *rpoB*-H445Y *Mtb* infection relative to littermate controls. Thus, during *rpoB*-H445Y *Mtb* infection, type I IFN signaling is protective in LysM-expressing cells, especially in the absence of IL-1 signaling, but detrimental in CD11c-expressing cells.

### NO production restricts intracellular *rpoB*-H445Y *Mtb* growth in murine macrophages

**Fig 4:**
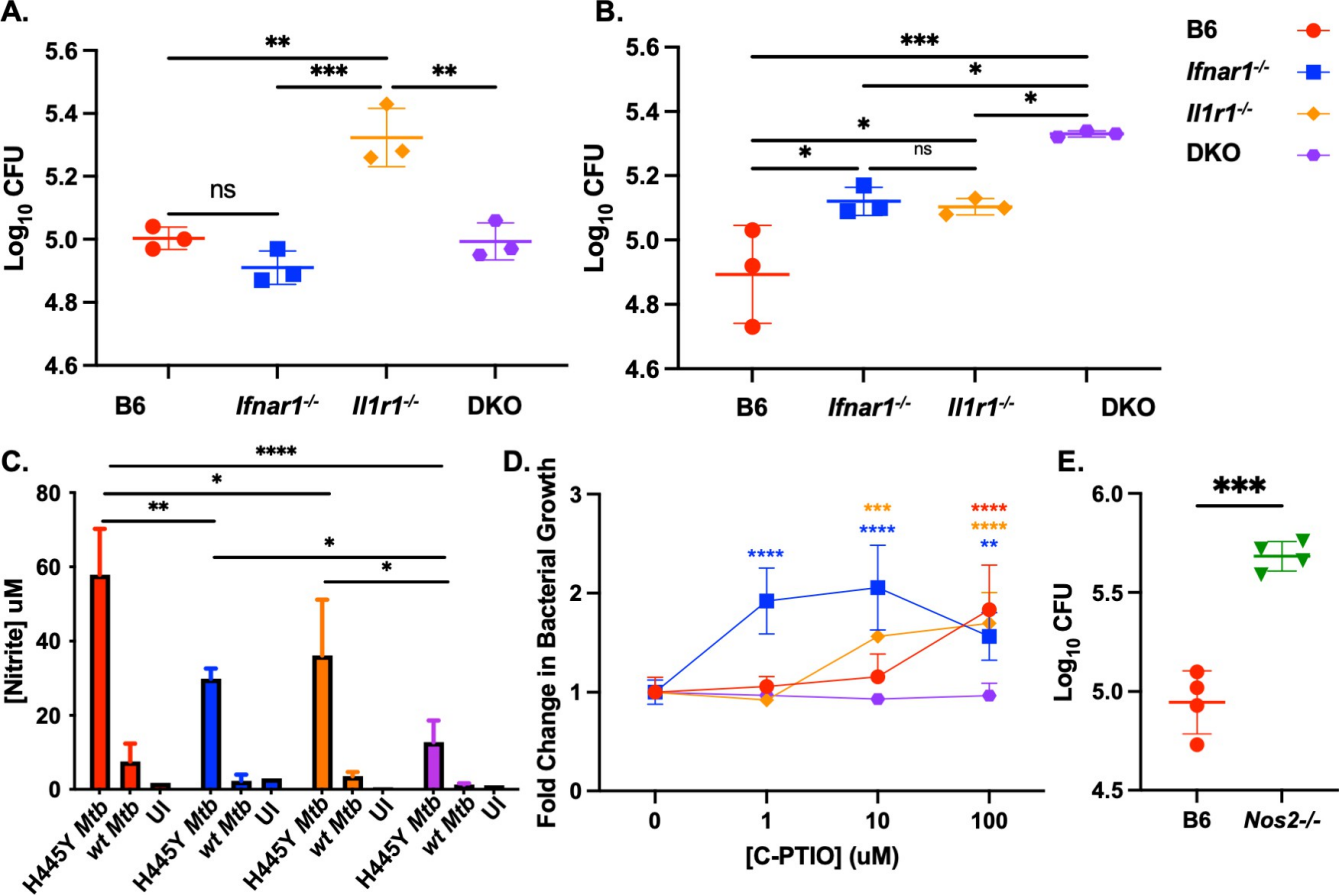
NO production restricts intracellular *rpoB*-H445Y *Mtb* growth in macrophages. Bone marrow-derived macrophages (BMDMs) from B6, *Ifnar1*^*-/-*^, *Il1r1*^*-/-*^, DKO mice were infected with either A) *wt Mtb* or B) *rpoB*-H445Y *Mtb*, and *Mtb* CFU was determined at 6 dpi. C) The concentration of nitrite in the supernatants of *wt* or *rpoB*-H445Y *Mtb* infected BMDMs was determined, along with uninfected controls. D) B6, *Il1r1*^*-/-*^, *Ifnar*^*-/-*^ and DKO BMDMs were infected with *rpoB*-H445Y *Mtb* and then treated with the indicated concentrations of an NO scavenger, Carboxy-PTIO (C-PTIO). Bacterial burden was determined on 6 dpi. Fold change in Mtb growth was determined relative to untreated and infected BMDMs. E) B6 and *Nos2*^*-/-*^ BMDMs were infected with *rpoB*-H445Y *Mtb*, and *Mtb* CFU was determined at 6 dpi. The data shown represent the means ± SD of three to four biological replicates per experiment. The data were evaluated for normality using the Shapiro-Wilk Test and passed (p-value > 0.05). One-way ANOVA and Two-way ANOVA, with Tukey’s multiple comparisons tests, were used for A-B and C-D, respectively. Unpaired two-tailed student’s t-test was used for E. Significant differences are indicated with asterisks (*, p-value ≤ 0.05; **, p-value ≤ 0.01; ***, p-value ≤ 0.001; ****, p-value ≤ 0.0001) by appropriate statistical tests. One of three independent experiments shown.

To address the importance of macrophages in producing and responding to type I IFN signals, we utilized bone marrow-derived macrophages (BMDMs) to elucidate the mechanistic role of type I IFN signaling in the absence of IL-1 signaling during *rpoB*-H445Y *Mtb* infection. As before [24], BMDMs lacking IL-1 signaling were more susceptible to *wt Mtb* infection at 6 dpi (Fig 4A). Additional loss of type I IFN signaling rescued this defect in the DKO BMDMs. In contrast, during *rpoB*-H445Y *Mtb* infection, loss of either type I IFN or IL-1 signaling led to an increase in intracellular *Mtb* growth, with loss of both (DKO) causing a further increase in *Mtb* growth (Fig 4B). We also assessed cell death dynamics during the BMDM infections (S5 Fig). Across the four genotypes, we find that BMDMs lacking only *Il1r1* exhibited increased cell death as seen with increased frequencies of cells at apoptotic (AnnexinV^hi^ 7AAD^lo^) and necrotic (AnnexinV^hi^ 7AAD^hi^) stages. While *wt Mtb* drove high levels of necrotic cell death early in infection, the *rpoB Mtb* mutant induced high levels of necrosis later during infection. Similarly, high levels of apoptosis were observed in *Il1r1*^*-/-*^ BMDMs only later during *rpoB* mutant *Mtb* infection. As both infections induced high levels of cell death in the *Il1r1*^*-/-*^ BMDMs, but not in the DKO BMDMs, these responses could be a general response to *Mtb* infection that is mediated by type I IFNs [34]. While these increased frequency of cell death in *Il1r1*^*-/-*^ BMDMs coincided with their inability to control *wt Mtb* infection, the same does not appear to be the case during *rpoB Mtb* mutant infection, suggesting a role for other host cellular processes to mediate protection against *rpoB*-H445Y *Mtb*.

Type I IFN signaling can drive nitric oxide (NO) production as part of its anti-bacterial functions [29]. Thus, we assessed the abundance of nitrite, the byproduct of NO generation, in the supernatants of *wt* or *rpoB*-H445Y *Mtb* infected BMDMs (Fig 4C). BMDMs from mice with functional type I IFN and IL-1 signaling produced increased NO in response to *rpoB*-H445Y *Mtb* infection when compared with other infected BMDMs. While there was a reduction in NO production in *rpoB*-H445Y *Mtb* infected BMDMs lacking either IL-1 or type I IFN signaling relative to infected B6 BMDMs, BMDMs from these two groups still produced more NO than their infected DKO counterparts. The stepwise decrease in NO production and the stepwise increase in CFU between B6 BMDMs to *Ifnar1*^-/-^ *and Il1r1*^*-/-*^ BMDMs to DKO BMDMs support the idea that NO production is associated with protection against *rpoB*-H445Y *Mtb* infection. To assess the role of NO in controlling *rpoB*-H445Y *Mtb* infection, we used carboxy-PTIO (C-PTIO), an NO scavenger, at varying concentrations (Fig 4D). Relative to untreated controls, the addition of 100 uM C-PTIO resulted in a loss of control in B6 BMDMs during *rpoB*-H445Y *Mtb* infection. In contrast, the addition of 1 uM and 10 uM of C-PTIO was sufficient to increase the susceptibility of *Ifnar1*^*-/-*^ and *Il1r1*^*-/-*^ BMDMs, respectively, while adding any amount of C-PTIO did not impact the course of infection in DKO BMDMs. This “minimum susceptibility” analysis suggests a stoichiometric role for NO in limiting *rpoB*-H445Y *Mtb* during infection. To more specifically assess the role of NO production in controlling *rpoB*-H445Y *Mtb* infection, we generated BMDMs from *Nos2*^*-/-*^ bone marrow and compared their ability to control bacterial replication relative to B6 BMDMs (Fig 4E). Similar to B6 BMDMs where NO production was inhibited by C-PTIO addition, BMDMs that were unable to produce NO due to lack of the inducible nitric oxide synthase were unable to control mycobacterial growth. Collectively, our data show that the protective mechanisms underlying type I IFN signaling are partially through NO production to restrict intracellular *rpoB*-H445Y *Mtb* replication.

## Discussion

IL-1 signaling is essential in the early immune response to *Mtb* infection [15,16]. However, the rifampin-resistant *rpoB*-H445Y *Mtb* bypass the need for IL-1 signaling, and the protective immune pathways in play have not been understood [24]. In this study, we show a protective role for type I IFN signaling in the absence of IL-1 signaling during infection with *rpoB*-H445Y *Mtb*. Additionally, the producers of type I IFN during infection are lung monocytes and recruited macrophages. During *rpoB*-H445Y *Mtb* infection, while LysM-expressing cells respond to the type I IFN signals to confer protection, type I IFN signaling in CD11c-expressing cells has a detrimental role. Finally, we show that type I IFN and IL-1 signaling cooperate to drive NO production to limit *rpoB*-H445Y *Mtb* replication. Together, our results demonstrate a protective role for type I IFN signaling during *rpoB*-H445Y *Mtb* infection, in contrast to its proposed detrimental role during *wt Mtb* infection. These results highlight fundamentally divergent host-pathogen interactions during closely related *Mtb* infections.

The impacts of type I IFN and IL-1 signaling in *wt Mtb* infection and on TB pathogenesis have been well studied [20,35,36]. In susceptible mouse models of TB such as C3Heb/FeJ and 129S2, type I IFNs facilitate the recruitment of *Mtb*-permissive cell types, drive neutrophil extracellular traps that impair *Mtb* control, and promote exacerbated immunopathology and disease [19,23,34,37]. Indeed, in human TB, type I IFNs and neutrophils are associated with and can predict the progression toward clinical TB diagnosis [22,38,39]. In models utilizing resistant mice such as B6, IL-1 signaling is necessary to control *Mtb* infection, especially through the production of eicosanoids, such as PGE2[15,16,18]. In these models, type I IFN signaling plays a detrimental role through antagonizing IL-1 production and signaling, limiting effective T cell immunity, and sensitizing infected macrophages towards death [17,18,34,37,40,41]. Our data also shows that lack of IL-1 signaling during *wt Mtb* infection results in increased frequencies of macrophage death *in vitro* as well as increased accumulation of neutrophils in the lungs *in vivo* and how these processes could be driven by type I IFN signaling. Heightening type I IFN induction through poly-IC treatment in B6 mice also led to increased *Mtb* lung burdens through recruitment of *Mtb*-permissive immune cells [42]. We have recently shown that type I IFN production is heightened during *rpoB*-H445Y *Mtb* infection, with type I IFN signaling leading to a suppressed immune response in the lungs of *rpoB*-H445Y *Mtb*-infected mice [25]. Correspondingly, lack of type I IFN signaling during *rpoB*-H445Y *Mtb* infection led to increased immune cell infiltration and activation and more inflammation, which is shown here as well. But, this more robust immune response in *Infar*^*-/-*^ mice led to better control of *rpoB*-H445Y *Mtb* and limited inflammation during the chronic stages of infection [25]. However, some studies have found a protective role for type I IFN signaling during *wt Mtb* infection [35,43]. For example, autocrine type I IFN signaling can promote macrophage control of *wt Mtb* infection, but *Mtb* has been shown to modulate host macrophage responses to limit potential protective autocrine type I IFN signaling, while promoting detrimental paracrine signaling [43]. Also, in a context without protective type II IFN signaling, additional loss of type I IFN signaling led to the double IFN knockout mice succumbing to *Mtb* infection earlier than either single IFN knockout mice [35]. Together, these studies suggest a complex role for type I IFN signaling where *wt Mtb* could be hijacking this host signal during infection to subvert protective immune responses such as IL-1 signaling.

In our work with the DR *Mtb* strains containing the *rpoB*-H445Y mutation, we have found that the impact of type I IFN and IL-1 signaling on *Mtb* infection are drastically changed. Previously, we found antagonism of IL-1 production by type I IFN signaling and heightened type I IFN production during infection with *rpoB-*H445Y *Mtb*. Yet, *rpoB-*H445Y *Mtb-*infected B6 mice were able to control infection, even without requiring IL-1 signaling [24]. In the current study, we find that type I IFN signaling pathway genes are induced in the lungs following *wt* or *rpoB*-H445Y *Mtb* infection. However, while decreased expression of genes induced by type I IFN was seen in infected DKO mice relative to their *Il1r1*^*-/-*^ counterparts, these transcriptional changes were associated with protective or detrimental host responses during *wt* or *rpoB*-H445Y *Mtb* infection, respectively. So, the difference in host control of these two infections could be driven by differences in which specific ISGs were induced as well as how those ISGs function in tandem with other upregulated cellular processes, such as different metabolic pathways and stress-related responses [24,44]. Thus, contextual cues could lead to diverse outcomes of type I IFN signaling during *Mtb* infections. Further work is needed to study how the *wt* and *rpoB*-H445Y *Mtb* strains drive differential expression of host genes and processes and how those pathways contribute to protective or detrimental effects of type I IFN signaling during infection.

Type I IFNs can be produced by many cell types, especially in response to microbial infection [36]. Following *rpoB*-H445Y *Mtb* infection, monocytes and RMs were the major producers of type I IFNs. Kotov *et al*. also recently investigated sources of type I IFNs during *wt Mtb* infection and found interstitial macrophages and pDCs to be dominant producers [45]. While we also found recruited monocyte and macrophage populations to be contributing to type I IFN production, we did not observe a significant population of pDCs in the lungs during infection, a cell type known to be a potent producer of type I IFNs [14,46], nor did we find IFN-producing non-immune epithelial cells [47]. These findings could be attributed to pDCs and epithelial cells acting earlier than 7 dpi or our approach not being sensitive enough to capture the pDC and epithelial cell populations. For example, Kotov *et al*. used a double fluorescent reporter sytem to identify all cells that have expressed *Ifnb1* at any point during infection, which would capture early or transient expression of type I IFNs. Thus, we cannot rule out a role for pDCs in our *rpoB*-H445Y *Mtb* infection model, which could be studied in follow-up work. Also, Kotov *et al*. carried out their studies using the *Mtb* Erdman strain, while we used the *Mtb* HN878 strain as our reference *wt* strain, and differences between these strains could impact the dynamics of type I IFN production by different cell types. Future experiments could also test whether myeloid cells directly infected with *Mtb* are type I IFN producers and how the kinetics of infection influences the kinetics of IFN production found in this study [48,49]. Additional insights can also be gained by utilizing newly emerging technologies such as spatial transcriptomics, to study localization of the IFN-producing cell types with respect to infected and other responding cell types [50–52].

Type I IFNs signal through the IFNAR1/IFNAR2 heterodimeric receptor, which is ubiquitously expressed [36]. Thus, depending on the cell type and other signaling processes, a wide variety of cellular responses can be induced, some that are protective and others that are detrimental. In the *rpoB*-H445Y *Mtb* infection model, even with functional IL-1 signaling, LysM-expressing cells required type I IFN signaling to drive a protective immune response, while type I IFN signaling was detrimental in CD11c-expressing cells. It should be noted that LysM is broadly expressed in a variety of immune cell types such as monocytes, macrophages, and granulocytes as well as some non-immune type II alveolar epithelial cells [33,53–55]. Our results show cell-specific effects of type I IFN signaling during *rpoB*-H445Y *Mtb* infection, but we can only speculate on the specific contribution of each cell type during infection. Neutrophils are a major cell type targeted by the LysM-Cre model and not affected in the CD11c-Cre model and, as noted above, have been associated with type I IFN signaling in other *Mtb* infections and shown here in *wt* HN878 *Mtb* infection. However, we saw no increases in numbers of neutrophils in either of the susceptible DKO mice or the *LysM-Cre*^*+*^ *Ifnar1*^*fl/fl*^ mice. While there could be differences in functionality between the neutrophils from protected and susceptible mice, such possibilities require follow-up investigation in the future. The consistent findings of increased abundance of activated RMs across susceptible mouse models, RMs as a source of type I IFN production, and a susceptible BMDM infection model suggest that these infiltrating macrophages are likely an immune subset contributing to a deficient immune response to *rpoB*-H445Y *Mtb* in the absence of type I IFN signaling.

On the other hand, CD11c expression in the lungs is restricted to AMs and DCs. As AMs are a common subset targeted by both Cre mouse models, it is likely that loss of type I IFN signaling in this subset does not play a role in *rpoB*-H445Y *Mtb* infection. However, our findings suggest a likely detrimental role for type I IFN signaling on DCs, which is supported by our previous study where we observed reduced BMDC migration *in vitro* and limited trafficking of *Mtb* to the draining lymph nodes *in vivo* during *rpoB*-H445Y *Mtb* infection [25]. We also previously found that global loss of *Ifnar* does not appear to impact *rpoB*-H445Y *Mtb* bacterial burden by 30 dpi, but *Ifnar*^*-/-*^ mice carry reduced bacterial loads during chronic stages of infection (≥ 50 dpi)[25]. Together, our data suggest that, in infected *Ifnar*^*-/-*^ mice, the deficiency in the ability of RMs to control *rpoB*-H445Y *Mtb* growth might be offset by a more robust adaptive immune response triggered by DCs that are not limited by type I IFN signaling, which also improves long-term control of *rpoB*-H445Y *Mtb* infection [56,57]. The susceptibility of the DKO mice to *rpoB*-H445Y *Mtb* infection and limited adaptive responses, relative to infected *Ifnar*^*-/-*^ mice, suggest that IL-1 signaling might play a role in the DC priming of the adaptive responses and is a mechanism contributing to control. The cell-cytokine specific contributions to protection against *rpoB*-H445Y *Mtb* should be explored in future studies.

In this study, we observed a role for NO in limiting *rpoB*-H445Y *Mtb* growth, with high levels of NO associated with control of *rpoB*-H445Y *Mtb* infection and limiting NO production resulted in increased *rpoB*-H445Y *Mtb* growth. This role for NO might be *Mtb* strain specific and not play a significant role during *wt Mtb* infection. Indeed, infection with *rpoB*-H445Y *Mtb* induced higher levels of NO than with the *wt Mtb* strain. Although we have not determined the mechanism behind the NO production, type I IFN and IL-1 signaling could function in tandem with another signal, such as TNF, to promote *Nos2* expression and NO production [58]. Similarly, on the bacterial side, PDIM or another virulence factor might contribute to suppression of NO production during *wt Mtb* infection, and this suppression might be lost during *rpoB*-H445Y *Mtb* infection due to differences in virulence factors between *wt Mtb* and and *rpoB*-H445Y *Mtb* strains [24]. Future work can aim to dissect the roles of host and pathogen factors in contributing to NO production. Studies in murine models suggest that NO contributes to limiting *Mtb* replication directly or indirectly, especially in macrophage subsets [59,60]. In primary human AM studies, NO induction has been mechanistically implicated in the killing of *Mycobacterium* species *in vitro* [61,62]. Thus, while NO production is elevated in murine cells it may not be reflective of *Mtb* control in human macrophages [63]. Our results show that both type I IFN and IL-1 signaling cooperate to induce production of NO in an additive manner. While type I IFN signaling has been more closely linked with NO production through the inducible nitric oxide synthase (*Nos2*) [43,64], it is possible that IL-1 could also drive NO production as in the case of Brucella infection [65]. Collectively, the data suggest that the dispensability of either signaling pathway during *in vivo rpoB*-H445Y *Mtb* infection is tied to compensatory NO production that is sufficient for protection.

An important question arising from this work is why *wt* and *rpoB*-H445Y *Mtb* are driving different infection outcomes. As we observe induction of similar pathways, albeit at different magnitudes, we hypothesize that differences in the expression of virulence factors between *wt* and *rpoB*-H445Y *Mtb* could influence the magnitude and timing of host cell responses. Concurrently, differential expression of virulence factors could impact the physiology of *rpoB*-H445Y *Mtb* and alter the response of the mutant *Mtb* to the pathways induced by type I IFN signaling, such as susceptibility to NO. We speculate that the location of the mutation in *rpoB*, which is a critical component of the bacterial transcription machinery, may result in broad transcriptional changes and expression of virulence factors [24,66]. We hypothesize that the increased production of long-chained PDIM in the *rpoB*-H445Y *Mtb* strain could be affecting the integrity of host phagosomes and lead to increased bacterial escape into the cytosol [67–70]. But, we have not thoroughly assessed all of the changes these DR mutations drive and how those changes are linked with the divergent host-pathogen interactions. A caveat of this study is that we cannot establish a causal link between the *rpoB*-H445Y mutation and the immune phenotypes observed in the study. The *rpoB*-H445Y *Mtb* strain used in this study was derived from the *wt Mtb* strain through antibiotic selection, and multiple independent DR *Mtb* isolates with the *rpoB*-H445Y mutation drove the same responses *in vitro* and *in vivo* [24]. However, a few additional mutations exist that separate the two strains outside of the *rpoB*-H445Y mutation, and we cannot rule out the possibility that additional mutations contribute to the differential host response [25]. The question of causality remains open and must be established in future work.

In summary, our studies have mechanistically shown how type I IFN signaling can drive protective or detrimental outcomes during *Mtb* infections and impact TB disease pathogenesis. Additionally, we uncover novel immune circuits through the use of a *rpoB*-H445Y *Mtb* strain with two mutually antagonistic signaling pathways acting in a compensatory but not redundant manner. Thus, our work highlights the necessity for greater investigation of host-pathogen interactions using a broader repertoire of *Mtb* strains and including drug-resistant isolates, which can reveal novel targets for future development of host-directed therapeutics.

## Materials and methods

### Ethics statement

Protocols involving the use of animals were approved by the Washington University in St. Louis or The University of Chicago Institutional Animal Care and Use Committee (IACUC) guidelines. All of the experiments were performed in accordance with the protocols.

### Mice

C57BL/6J (B6), *Ifnar1*^−/−^, *Il1r1*^*-/-*^, *Nos2*^*-/-*^, and *Ifnb*^*mob*^ mice on the B6 background were purchased from The Jackson Laboratory (Bar Harbor, ME). *Ifnar1*^*-/-*^ *Il1r1*^*-/-*^ mice were generated by crossing *Ifnar1*^−/−^ and *Il1r1*^*-/-*^ mice. *LysM*-Cre *Ifnar1*^*fl/fl*^ and *Cd11c*-Cre *Ifnar1*^*fl/fl*^ mice were generously provided by the Baldridge Lab (Washington University in St. Louis). In some experiments, *LysM-*Cre^+^ and *LysM-*Cre^-^ *Ifnar1*^*fl/fl*^ mice were administered sterile PBS or a-IL1R blocking antibody (BioXCell) at 100 ug/mouse in 200uL every other day intraperitoneally (IP) starting at -1 dpi. All mice were maintained in the animal facility at Washington University in St. Louis and bred in house. Experimental mice were age and sex matched and infected between the ages of 6 and 8 weeks. All mice were maintained and used in accordance with the approved Washington University in St. Louis Institutional Animal Care and Use Committee guidelines.

### Generation of BMDMs

Bone marrow derived macrophages (BMDMs) were generated as previously described [11,24]. Briefly, bone marrow cells were collected from the tibia and femur of B6 mice and cultured in complete Dulbecco’s modified eagle’s medium (cDMEM) with 20 ng/mL of recombinant granulocyte-macrophage colony-stimulating factor (GM-CSF). Cells were cultured at 37°C in 5% CO_2_ and supplemented with media on day 3. On day 7, floating cells were taken up and discarded, and adherent cells were collected as BMDMs.

### *Mtb* infections

*Mtb* strain HN878 (*wt*) was obtained from BEI resources (Manassas, VA) under National Institutes of Health contract AI-75320. Independent rifampicin resistant *Mtb* colonies (biological replicates) were selected from rifampicin (2 μg/ml) containing 7H11 agar plates [3]. The sequences of *rpoB* were confirmed by Sanger sequencing (Genewiz), and *Mtb* stocks were created for further experimentation. The whole genomes of all *Mtb* strains were previously sequenced, with all SNPs between both, *wt* and *rpoB*-H445Y, *Mtb* strains are published [25]. All *Mtb* strains were cultured in Proskauer Beck medium supplemented with 0.05% Tween 80 and frozen at −80°C while in mid-log phase prior to use in mouse and macrophage infections. Colony forming units (CFU) of the bacterial stocks were determined through serial dilutions on 7H11 agar plates. Mice were aerosol infected with low doses (~100 CFU) of indicated *Mtb* strains in sterile PBS using a Glas-col nebulizer [71]. *Mtb* bacterial burden/organ was quantitated by plating serial dilutions of homogenized lung tissue on 7H11 agar plates (BD Biosciences, Franklin Lakes, NJ). Plates were incubated for 2–3 weeks at 37°C, and the number of colonies were counted. *In vitro* infections were carried out at a multiplicity of infection (MOI) of 1 in antibiotic free media for six days. In some experiments, macrophages were treated with indicated amounts of carboxy-PTIO (C-PTIO) four hours after the start of infection. Bacterial burden in each well was determined through serial plating of supernatant and lysed macrophages after six days post infection.

### Flow cytometry

Lungs were perfused with heparin in PBS, minced, digested with DNAse/collagenase, lysed for red blood cells, and pressed through a 70 μm filter to generate a single cell suspension [71]. For quantification of cytokine responses, single cell suspensions were stimulated *ex vivo* with purified *Mtb* antigens ESAT6 and Ag85B (10 μg/mL) overnight followed by a five hour stimulation with brefeldin and monensin (BD Biosciences) as previously described [9,10]. Cells were treated with Fc Block (CD16/CD32,2.4G2, Tonbo Biosciences) and stained with appropriate fluorochrome-labeled specific antibodies or isotype control antibodies. Intracellular cytokine staining was performed using the BD Cytofix/Cytoperm kit (BD Biosciences). Mouse antibodies used include CD11b (M1/70; Tonbo Biosciences), CD11c (HL3; BD Biosciences), Gr-1 (RB6–8C5, eBioscience), SiglecF (E50-2440; BD Biosciences), CD64 (X54-5/7.1; Biolegend), GFP polyclonal antibody (Cat#A21311; Thermo Fisher) or rabbit IgG isotype control (Catalog # 11-4614-80), CD3 (500A2; BioLegend), CD4 (RM4–5; BD Biosciences), CD44 (IM7; Tonbo Biosciences), IFN-*γ* (XMG1.2; Tonbo Biosciences) or isotype control IgG1, k (A85-1; BD Biosciences), CD8 (53–6.7; BD Biosciences), and MHC Class II (M5/114.15.2; Tonbo Biosciences).

Cells were processed with the Becton Dickinson (BD) Fortessa X-20 flow cytometer using FACS Diva software, or the BD FACSJazz flow cytometer using FACS software (BD). Flow cytometry experiments were analyzed using FlowJo (Tree Star Inc.). As before [11,24,72], neutrophils were defined as CD11b^+^CD11c^-^Gr-1^hi^, monocytes were defined as CD11b^+^CD11c^-^Gr-1^med^ cells, and recruited macrophages (RMs) were defined as CD11b^+^CD11c^-^Gr-1^low^ cells. Myeloid dendritic cells (mDCs) were defined as CD11b^+^CD11c^+^ cells and alveolar macrophages (AMs) were defined as CD11b^-^CD11c^+^SiglecF^hi^CD64^hi^. T cells were defined as CD3^+^CD4^+^ or CD3^+^CD8^+^ cells. Total numbers of cells within each gate were back-calculated based on cell counts/individual sample.

For the determination of frequencies of apoptotic and necrotic cells, BMDMs were collected at the indicated timepoints and stained with Annexin V (PE) and 7AAD as per the instructions of the PE Annexin V Apoptosis Detection Kit (559763, BD Pharmigen), prior to processing on the X-20 flow cytometer.

### Histology

Lung lobes were perfused with 10% neutral buffered formalin and embedded in paraffin. Formalin-fixed paraffin-embedded (FFPE) lung sections were stained with hematoxylin and eosin (H&E) and inflammatory features were evaluated by light microscopy. Inflammatory lesions were outlined with the automated tool of the Zeiss Axioplan 2 microscope (Carl Zeiss) and the total inflammatory area in each lung lobe was measured.

### Protein determination

BMDMs were either stimulated with *Mtb* antigens for 1 day or left unstimulated, lysed in RIPA Lysis Buffer (Santa Cruz Biotechnology), and heat denatured in reducing sample buffer (Thermo Fisher Scientific). Proteins were separated in 4% to 20% polyacrylamide gradient gels (Bio-Rad) and transferred onto PVDF membranes. Membranes were probed with primary antibodies against IFNAR1 (R&D, Cat#AF3039), and GAPDH (Santa Cruz Biotechnology, Cat# SC-25778) followed by incubation with HRP-conjugated anti–rabbit IgG secondary antibody (Santa Cruz Biotechnology) and developed by Clarity Western ECL Substrate (Bio-Rad).

### RNA isolation, sequencing, and processing

In brief, lung tissue was homogenized in RLT buffer, and total RNA was extracted with RNeasy Mini Kit (Qiagen) as previously described [11]. Total RNA integrity was determined using Agilent Bioanalyzer or 4200 Tapestation. Library preparation was performed with 5 to 10ug of total RNA with a Bioanalyzer RIN score greater than 8.0. Ribosomal RNA was removed by poly-A selection using Oligo-dT beads (mRNA Direct kit, Life Technologies). mRNA was then fragmented in reverse transcriptase buffer and heating to 94 degrees for 8 minutes. mRNA was reverse transcribed to yield cDNA using SuperScript III RT enzyme (Life Technologies, per manufacturer’s instructions) and random hexamers. A second strand reaction was performed to yield ds-cDNA. cDNA was blunt ended, had an A base added to the 3’ ends, and then had Illumina sequencing adapters ligated to the ends. Ligated fragments were then amplified for 12–15 cycles using primers incorporating unique dual index tags. Fragments were sequenced on an Illumina NovaSeq-6000 using paired end reads extending 150 bases. RNA-seq reads were then aligned and quantitated to the Ensembl release 101 primary assembly with an Illumina DRAGEN Bio-IT on-premise server running version 3.9.3–8 software (GTAC MGI).

The read count data of each gene were generated using featureCounts from Rsubread package [73] with default parameter settings. Significantly differentially expressed genes across different groups were identified using DESeq2, version 1.30.1[74], with default settings and a minimum p-value significance threshold of 0.05 (after FDR correction for the number of tests)[75]. Pathway enrichment analysis among differentially expressed gene sets of interest was performed for Reactome pathways using ReactomePA [76] with p-value ≤ 0.05 after FDR correction. Gene set enrichment analysis was also performed among differentially expressed gene sets of interest with hallmark gene sets for *Mus musculus* using fgsea [77] with eps set to zero to estimate p-value more accurately and p-value ≤ 0.05 after FDR correction. Additionally, several R packages were used including stringr [78], pheatmap [79], ggplot2[80], and org.Hs.eg.db [81] for data processing and generate figures.

### Statistical analysis

All datasets were evaluated for normality using the Shapiro-Wilk Test. Differences between the means of more than two groups were analyzed using One-way ANOVA or Two-way ANOVA, where applicable, with Tukey’s post-tests for normally distributed distributions or Kruskal Wallis Test with Dunn’s multiple comparisons tests. Differences between the means of two groups were analyzed by the unpaired two-tailed student’s t-test. All statistical analyses were done in GraphPad Prism 9. A p-value < 0.05 was considered significant. The data points across the figures represent the mean (±SD or +SEM) of values as noted. *, p≤0.05; **, p≤0.01; ***, p≤0.001; ****, p≤0.0001; ns-not significant (p>0.05). All experiments were replicated for reproducibility.

## Supporting information

S1 Fig Loss of control of *wt Mtb* infection is associated with increased inflammatory damage and increased abundance of some myeloid subsets. C57Bl/6 (B6), *Ifnar1*^*-/-*^, *Il1r1*^*-/-*^, and *Ifnar1*^*-/-*^ *Il1r1*^*-/-*^ [double knockout (DKO)] mice were aerosol infected with a low dose of *wt Mtb* and sacrificed after 30 dpi. A) Formalin-fixed, paraffin embedded (FFPE) lung sections were stained with hematoxylin and eosin (H&E), and the total inflammatory areas were quantified after *wt Mtb* infection at 30 days post infection (dpi). B) Total numbers of Major Histocompatibility Complex Class II^hi^ (MHCII^hi^) recruited macrophages (RMs), myeloid dendritic cells (mDCs) MHCII^hi^, neutrophils, MHCII^hi^ alveolar macrophages (AMs), and MHCII^hi^ monocytes were determined via flow cytometry. The numbers of C) CD4^+^ CD44^hi^ IFNg^+^ T cells and D) CD8^+^ CD44^hi^ IFNg^+^ T cells were also determined by flow cytometry following *ex* vivo stimulation with purified *Mtb* antigens ESAT6 and Ag85B. The data shown represent the means ± SD of four to seven mice per experiment. The data were evaluated for normality using the Shapiro-Wilk Test and passed (p-value > 0.05). One-way ANOVA, with Tukey’s multiple comparisons tests, were used for A-D. Significant differences are indicated with asterisks (*, p-value ≤ 0.05; ns, p-value > 0.05) by appropriate statistical tests. One of three independent experiments shown.
(TIF)

S2 Fig Loss of control of *rpoB*-H445Y *Mtb* infection is not associated with changes in other myeloid or T cell subsets. C57Bl/6 (B6), *Ifnar1*^*-/-*^, *Il1r1*^*-/-*^, and *Ifnar1*^*-/-*^ *Il1r1*^*-/-*^ [double knockout (DKO)] mice were aerosol infected with a low dose of *rpoB*-H445Y *Mtb* and sacrificed after 30 dpi. Total numbers of A) neutrophils, B) Major Histocompatibility Complex Class II^hi^ (MHCII^hi^) monocytes, and C) MHCII^hi^ alveolar macrophages (AMs) were determined via flow cytometry. The numbers of D) CD4^+^ CD44^hi^ IFNg^+^ T cells and E) CD8^+^ CD44^hi^ IFNg^+^ T cells were also determined by flow cytometry following *ex* vivo stimulation with purified *Mtb* antigens ESAT6 and Ag85B. The data shown represent the means ± SD of five to nine mice per experiment. The data were evaluated for normality using the Shapiro-Wilk Test and passed (p-value > 0.05). One-way ANOVA, with Tukey’s multiple comparisons tests, were used for A-E. Significant differences are indicated with asterisks (*, p-value ≤ 0.05; **, p-value ≤ 0.01; ***, p-value ≤ 0.001; ****, p-value ≤ 0.0001) by appropriate statistical tests. One of three independent experiments shown.
(TIF)

S3 Fig Type I IFN signatures are upregulated in B6 and IL-1 knockout mice infected with either *wt* or *rpoB*-H445Y *Mtb*. C57Bl/6 (B6), *Ifnar1*^*-/-*^, *Il1r1*^*-/-*^, and *Ifnar1*^*-/-*^ *Il1r1*^*-/-*^ (DKO) mice were aerosol infected with a low dose of *wt* or *rpoB*-H445Y *Mtb* and sacrificed at 14 dpi. RNA was extracted from homogenized lung tissue and sequenced. The expression of genes in the type I IFN pathway in bulk lung cells from DKO mice over bulk lung cells from the *wt* mice is shown (top), DKO mice over *Il1r1-/-* mice (middle), and *Ifnar1-/-* over *wt* mice (bottom) after A) *wt* or B) *rpoB*-H445Y *Mtb* infection with annotated pathways (n = 4–6). Gene set enrichment analysis was done using an fgsea R package as noted in Methods. The pathways are enriched in the second group of each comparison.
(TIF)

S4 Fig Increased susceptibility of *LysM-Cre*^*+*^ *Ifnar1*^*fl/fl*^ mice to *rpoB*-H445Y *Mtb* infection is associated with exacerbated immunopathology. As described in Fig 3C, *LysM-Cre*^*+*^ *Ifnar1*^*fl/fl*^ mice and littermate controls were aerosol infected with a low dose of *rpoB*-H445Y *Mtb* and administered a-IL1R or isotype antibodies i.p. every other day, starting one day before infection, and sacrificed at 28 dpi. A) FFPE lung sections that were H&E stained were prepared and the inflammatory areas were quantified at 28 dpi. Representative histological images are shown, with quantification of total inflammatory area divided by total lung area for each lobe depicted below. B) Through flow cytometry, total numbers of MHCII^hi^ RMs and mDCs were calculated in the lungs at 28 dpi with the *rpoB*-H445Y *Mtb* strain. The data shown represent the means ± SD of five to nine mice per experiment. The data were evaluated for normality using the Shapiro-Wilk Test and passed (p-value > 0.05). One-way ANOVA, with Tukey’s multiple comparisons tests, were used for A and B. Significant differences are indicated with asterisks (*, p-value ≤ 0.05; **, p-value ≤ 0.01; ***, p-value ≤ 0.001; ****, p-value ≤ 0.0001) by appropriate statistical tests. One of two independent experiments shown.
(TIF)

S5 Fig Cell death dynamics of *rpoB*-H445Y *Mtb*-infected BMDMs are influenced by genotype but are not directly correlated with susceptibility. BMDMs from B6, *Ifnar1*^*-/-*^, *Il1r1*^*-/-*^, DKO mice were infected with either *wt Mtb* (circles) or *rpoB*-H445Y *Mtb* (squares). At indicated timepoints post infection, BMDMs were collected, stained with Annexin V and 7AAD, per manufacturer’s instruction, and processed using flow cytometry. Uninfected controls were also included. The frequencies of A) apoptotic (7AAD^lo^ AnnexinV^hi^) and B) necrotic (7AAD^hi^ AnnexinV^hi^) BMDMs were quantified. The data shown represent the means ± SD of three to four biological replicates per experiment. The data were evaluated for normality using the Shapiro-Wilk Test and passed (p-value > 0.05). Two-way ANOVA, with Tukey’s multiple comparisons tests, was used for A and B. Significant differences are indicated with asterisks (*, p-value ≤ 0.05; **, p-value ≤ 0.01; ***, p-value ≤ 0.001; ****, p-value ≤ 0.0001) by appropriate statistical tests. One of two independent experiments shown.
(TIF)

S1 Data This file contains the source data used to generate every figure in this manuscript. Each tab of the Excel file corresponds to a specific figure panel (main and supplemental) and is labeled as such.
(XLSX)
