# Supplementary figures and images for: A protective role for type I interferon signaling following infection with Mycobacterium tuberculosis carrying the rifampicin drug resistance-conferring RpoB mutation H445Y
Source: PLoS Pathog. 2024 Apr 11;20(4):e1012137. doi: 10.1371/journal.ppat.1012137 (PMC11037539; doi:10.1371/journal.ppat.1012137)

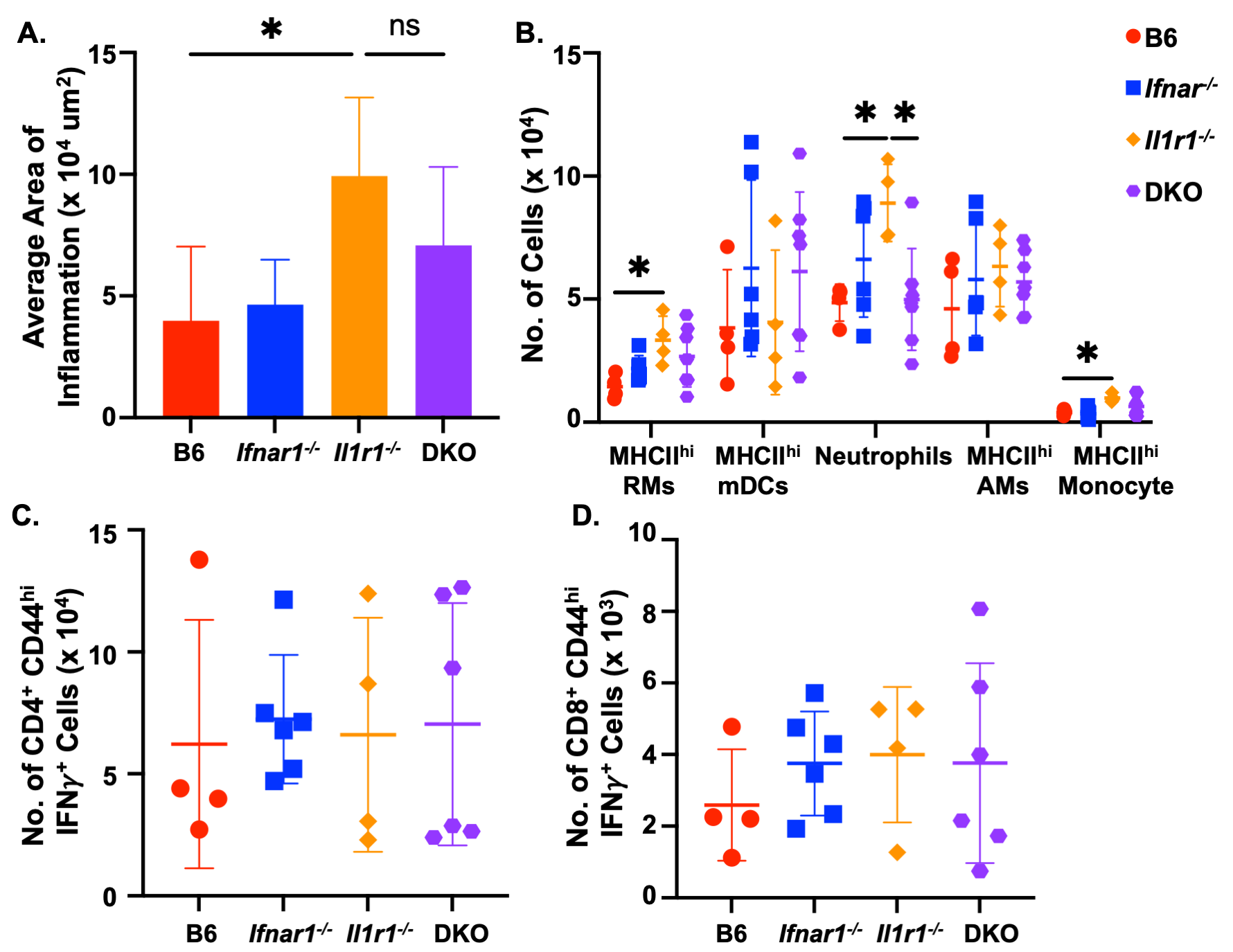

Supplement: S1 Fig — C57Bl/6 (B6), Ifnar1-/-, Il1r1-/-, and Ifnar1-/- Il1r1-/- [double knockout (DKO)] mice were aerosol infected with a low dose of wt Mtb and sacrificed after 30 dpi. A) Formalin-fixed, paraffin embedded (FFPE) lung sections were stained with hematoxylin and eosin (H&E), and the total inflammatory areas were quantified after wt Mtb infection at 30 days post infection (dpi). B) Total numbers of Major Histocompatibility Complex Class IIhi (MHCIIhi) recruited macrophages (RMs), myeloid dendritic cells (mDCs) MHCIIhi, neutrophils, MHCIIhi alveolar macrophages (AMs), and MHCIIhi monocytes were determined via flow cytometry. The numbers of C) CD4+ CD44hi IFNg+ T cells and D) CD8+ CD44hi IFNg+ T cells were also determined by flow cytometry following ex vivo stimulation with purified Mtb antigens ESAT6 and Ag85B. The data shown represent the means ± SD of four to seven mice per experiment. The data were evaluated for normality using the Shapiro-Wilk Test and passed (p-value > 0.05). One-way ANOVA, with Tukey’s multiple comparisons tests, were used for A-D. Significant differences are indicated with asterisks (*, p-value ≤ 0.05; ns, p-value > 0.05) by appropriate statistical tests. One of three independent experiments shown. (TIF) [file ppat.1012137.s001.tif]

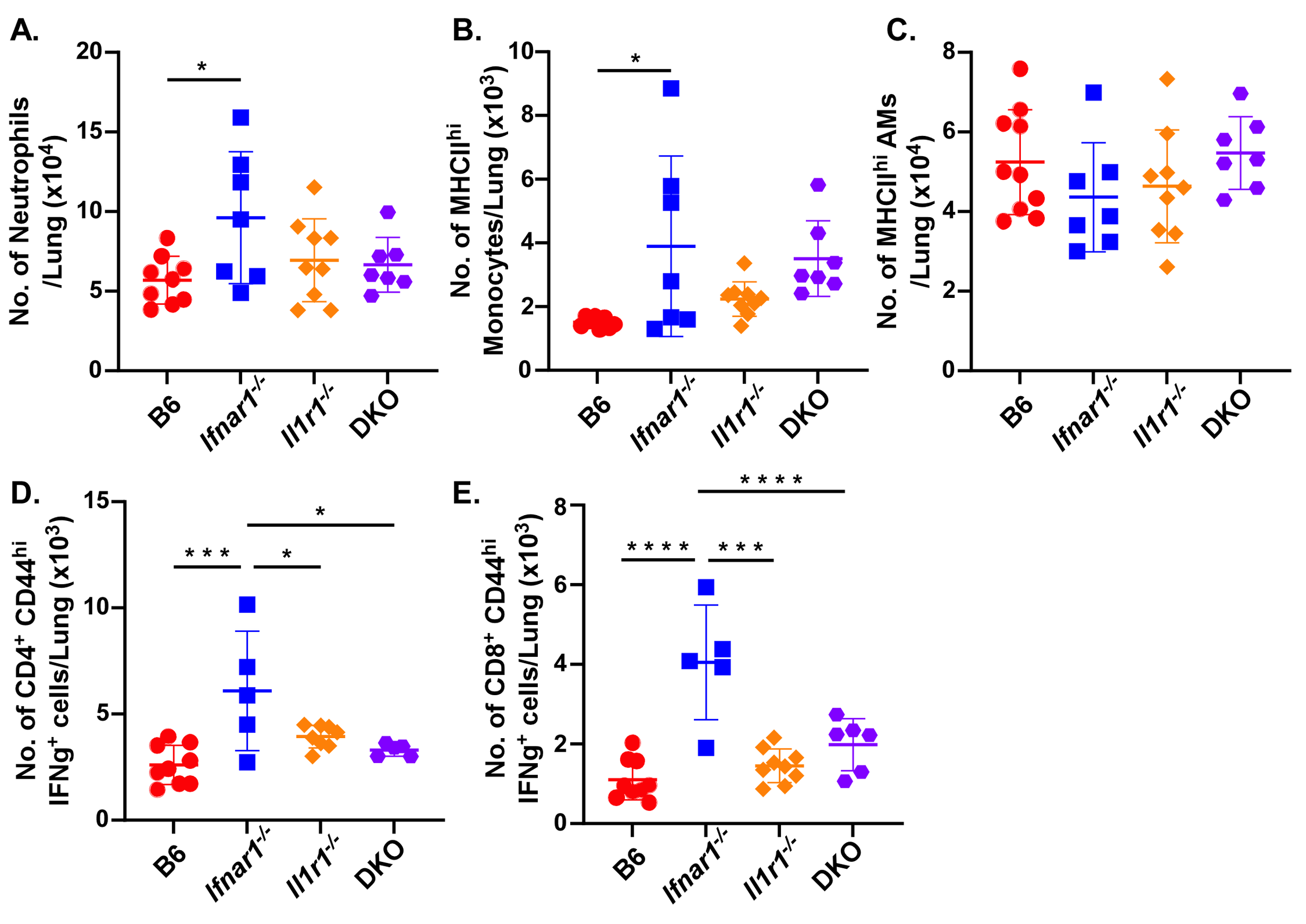

Supplement: S2 Fig — C57Bl/6 (B6), Ifnar1-/-, Il1r1-/-, and Ifnar1-/- Il1r1-/- [double knockout (DKO)] mice were aerosol infected with a low dose of rpoB-H445Y Mtb and sacrificed after 30 dpi. Total numbers of A) neutrophils, B) Major Histocompatibility Complex Class IIhi (MHCIIhi) monocytes, and C) MHCIIhi alveolar macrophages (AMs) were determined via flow cytometry. The numbers of D) CD4+ CD44hi IFNg+ T cells and E) CD8+ CD44hi IFNg+ T cells were also determined by flow cytometry following ex vivo stimulation with purified Mtb antigens ESAT6 and Ag85B. The data shown represent the means ± SD of five to nine mice per experiment. The data were evaluated for normality using the Shapiro-Wilk Test and passed (p-value > 0.05). One-way ANOVA, with Tukey’s multiple comparisons tests, were used for A-E. Significant differences are indicated with asterisks (*, p-value ≤ 0.05; **, p-value ≤ 0.01; ***, p-value ≤ 0.001; ****, p-value ≤ 0.0001) by appropriate statistical tests. One of three independent experiments shown. (TIF) [file ppat.1012137.s002.tif]

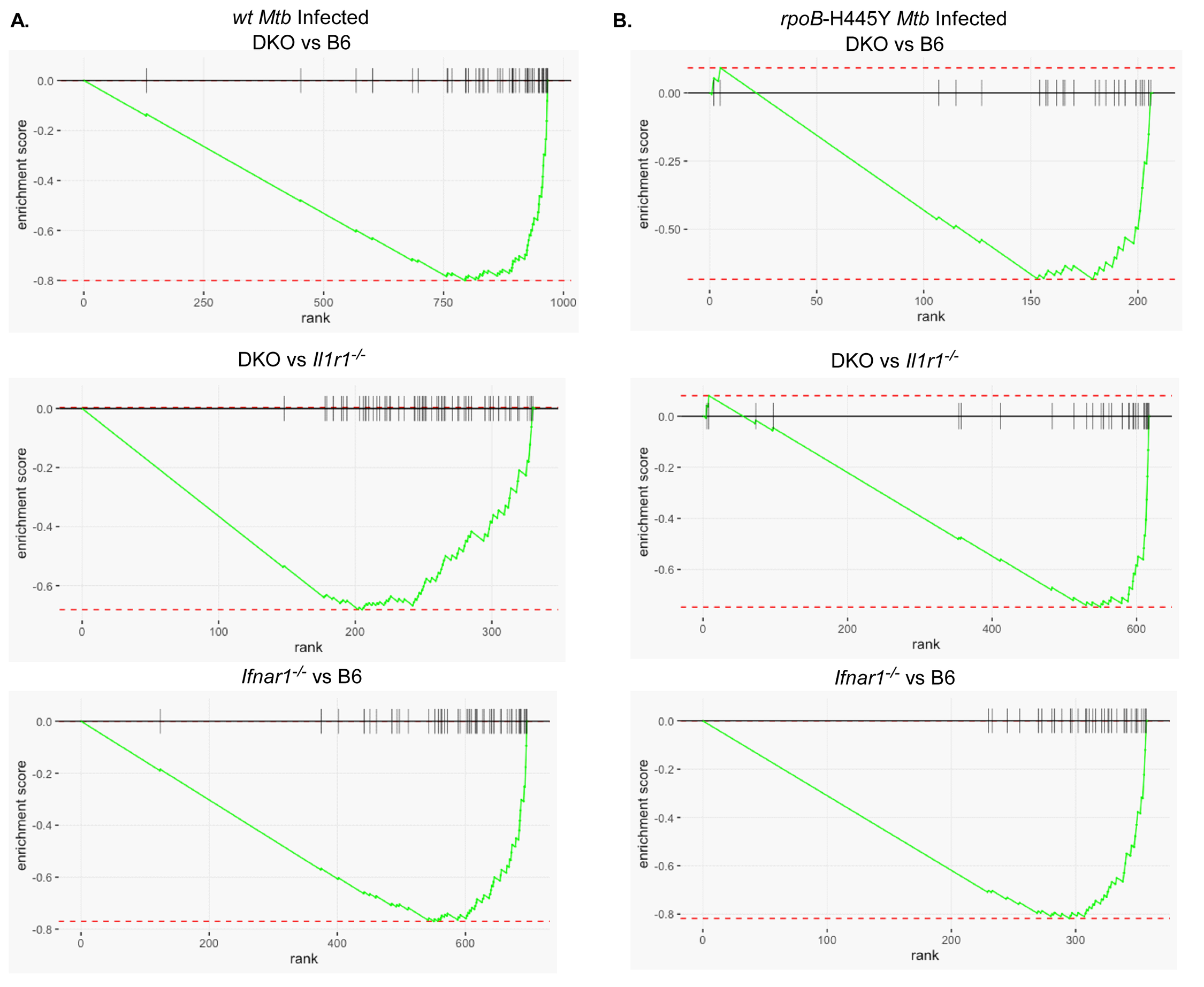

Supplement: S3 Fig — C57Bl/6 (B6), Ifnar1-/-, Il1r1-/-, and Ifnar1-/- Il1r1-/- (DKO) mice were aerosol infected with a low dose of wt or rpoB-H445Y Mtb and sacrificed at 14 dpi. RNA was extracted from homogenized lung tissue and sequenced. The expression of genes in the type I IFN pathway in bulk lung cells from DKO mice over bulk lung cells from the wt mice is shown (top), DKO mice over Il1r1-/- mice (middle), and Ifnar1-/- over wt mice (bottom) after A) wt or B) rpoB-H445Y Mtb infection with annotated pathways (n = 4–6). Gene set enrichment analysis was done using an fgsea R package as noted in Methods. The pathways are enriched in the second group of each comparison. (TIF) [file ppat.1012137.s003.tif]

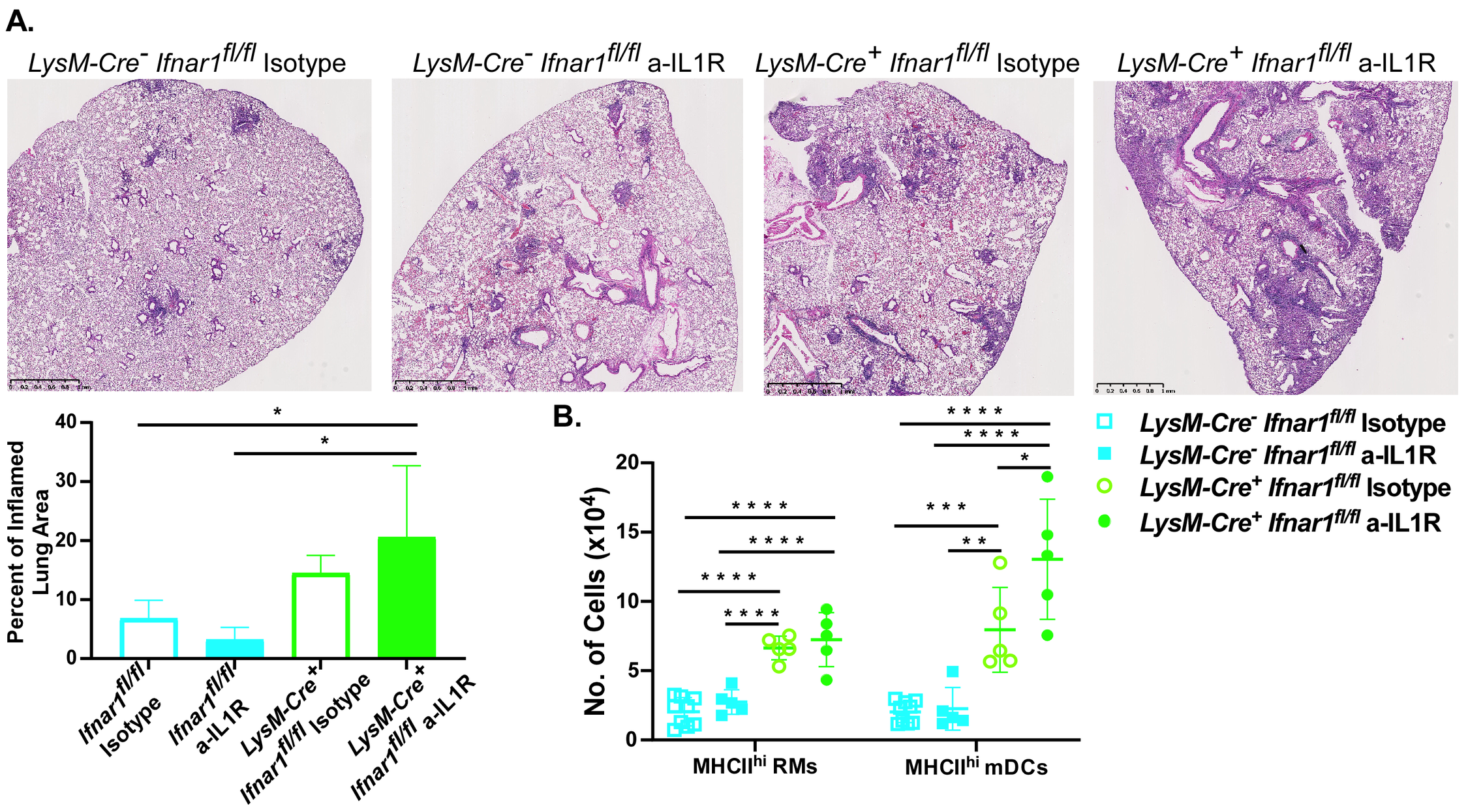

Supplement: S4 Fig — As described in Fig 3C, LysM-Cre+ Ifnar1fl/fl mice and littermate controls were aerosol infected with a low dose of rpoB-H445Y Mtb and administered a-IL1R or isotype antibodies i.p. every other day, starting one day before infection, and sacrificed at 28 dpi. A) FFPE lung sections that were H&E stained were prepared and the inflammatory areas were quantified at 28 dpi. Representative histological images are shown, with quantification of total inflammatory area divided by total lung area for each lobe depicted below. B) Through flow cytometry, total numbers of MHCIIhi RMs and mDCs were calculated in the lungs at 28 dpi with the rpoB-H445Y Mtb strain. The data shown represent the means ± SD of five to nine mice per experiment. The data were evaluated for normality using the Shapiro-Wilk Test and passed (p-value > 0.05). One-way ANOVA, with Tukey’s multiple comparisons tests, were used for A and B. Significant differences are indicated with asterisks (*, p-value ≤ 0.05; **, p-value ≤ 0.01; ***, p-value ≤ 0.001; ****, p-value ≤ 0.0001) by appropriate statistical tests. One of two independent experiments shown. (TIF) [file ppat.1012137.s004.tif]

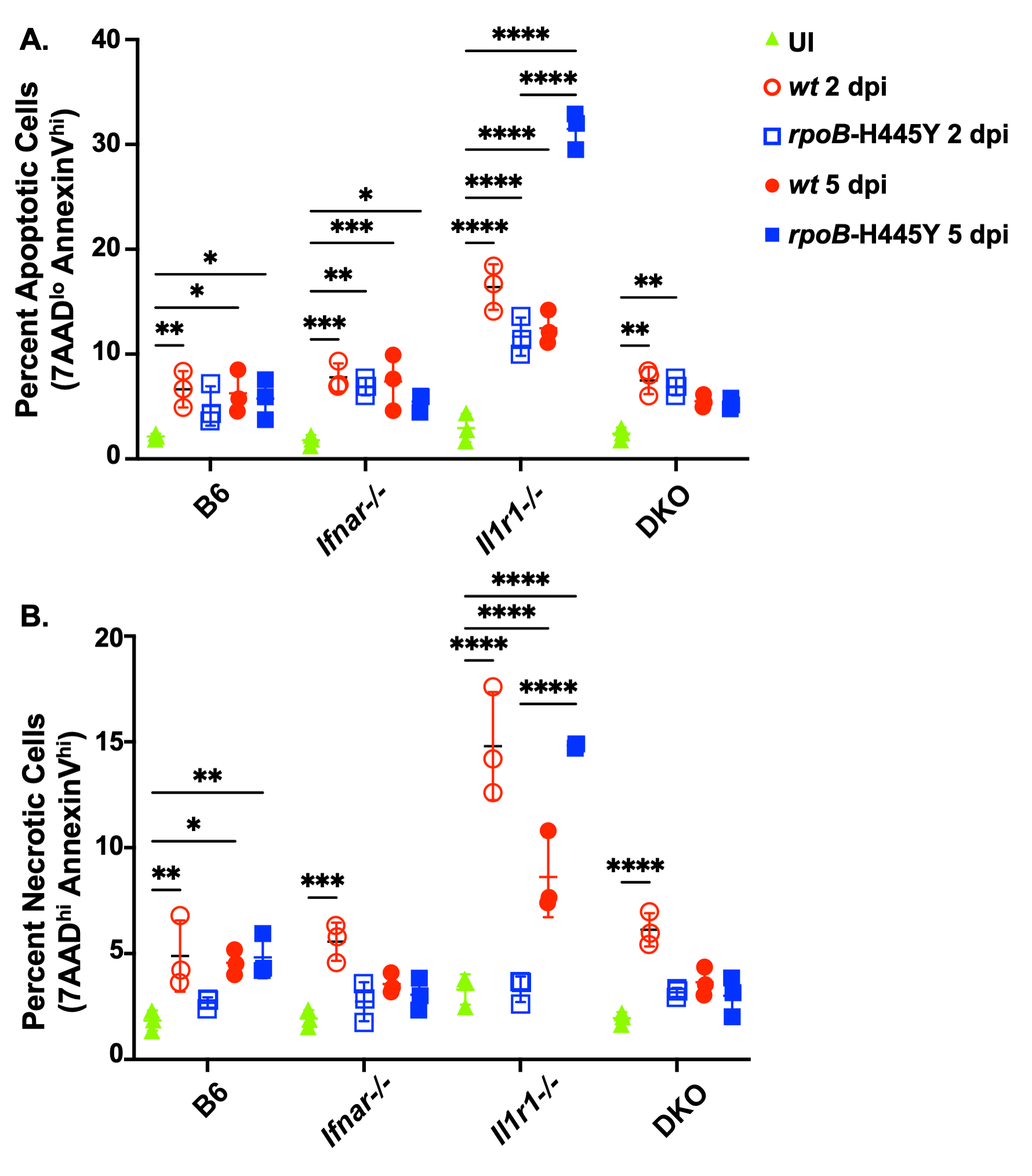

Supplement: S5 Fig — BMDMs from B6, Ifnar1-/-, Il1r1-/-, DKO mice were infected with either wt Mtb (circles) or rpoB-H445Y Mtb (squares). At indicated timepoints post infection, BMDMs were collected, stained with Annexin V and 7AAD, per manufacturer’s instruction, and processed using flow cytometry. Uninfected controls were also included. The frequencies of A) apoptotic (7AADlo AnnexinVhi) and B) necrotic (7AADhi AnnexinVhi) BMDMs were quantified. The data shown represent the means ± SD of three to four biological replicates per experiment. The data were evaluated for normality using the Shapiro-Wilk Test and passed (p-value > 0.05). Two-way ANOVA, with Tukey’s multiple comparisons tests, was used for A and B. Significant differences are indicated with asterisks (*, p-value ≤ 0.05; **, p-value ≤ 0.01; ***, p-value ≤ 0.001; ****, p-value ≤ 0.0001) by appropriate statistical tests. One of two independent experiments shown. (TIF) [file ppat.1012137.s005.tif]
